# Types of psychosocial job demands and adverse events due to dental mismanagement: a cross sectional study

**DOI:** 10.1186/1472-6831-7-3

**Published:** 2007-04-04

**Authors:** Akizumi Tsutsumi, Katsura Umehara, Hiroshi Ono, Norito Kawakami

**Affiliations:** 1 University of Occupational and Environmental Health, Occupational Health Training Center, 1-1 Iseigaoka, Yahatanishi-ku, Kitakyushu 807-8555, Japan; 2 Okayama University Graduate School of Medicine, Dentistry and Pharmaceutical Sciences, Hygiene & Preventive Medicine, 5-1 Shikata-cho,2-chome, Okayama 700-8558, Japan; 3 Ono Dental Clinic, 1018-15 Niina, Takase-machi, Mitoyo-shi, Kagawa 767-0002, Japan; 4 Department of Mental Health, Department of Psychiatric Nursing, Tokyo University Graduate School of Medicine, 7-3-1 Hongo, Bunkyo-ku, Tokyo 113-0033, Japan

## Abstract

**Background:**

A harsh work environment including psychosocial job demands might cause adverse events due to medical mismanagement, but the association has not been explored. The purpose of the study was to investigate whether some types of psychosocial job demands are associated with adverse events due to dental mismanagement experienced by general dental practitioners.

**Methods:**

A self-administered questionnaire was mailed to members of a local branch of the Japan dental association. A total of 261 dental practitioners responded anonymously (response rate 53%). Psychosocial job demands were measured by a Japanese version of the Copenhagen Psychosocial Questionnaire, which comprises five sub-scales: quantitative demands, cognitive demands, emotional demands, demands for hiding emotions, and sensorial demands. The outcome was defined according to whether the respondent's patients experienced one of the following adverse events due to dental mismanagement at least once during the previous one year: dropping of dental instrument or broken injection needle, soft tissue or nerve injury, accidental bleeding, loss of a tooth root into the maxillary sinus, and emphysema. Associations between each demand index and experience of adverse events were examined by logistic regression analyses adjusting for potential confounders.

**Results:**

Emotional demands and sensorial demands were significantly associated with the experience of adverse events (odds ratio = 3.9 for each). Other than the indices, male gender, younger age, practice alone, many dental chairs (five or more), and many patients (30 or more per day) were the risks. Working hours per week and number of paramedical staff had no significant associations.

**Conclusion:**

Emotional and sensorial job demands are a potential target for the reduction of adverse events due to dental mismanagement.

## Background

Injury or complications in patients undergoing medical mismanagement has become a significant social concern, as the consequences are often very serious [1]. Thus, the benefits of preventing such adverse events are large for the appropriate health services.

It has been reported that dental practitioners experience higher levels of occupational stress than general working populations [2-5]. As classifications of dental work pressure, rising patient expectations, aggression exhibited by some patients in the practice, the risk of cross-infection, litigation and working as a team member are recently identified stressors [6]. Furthermore, dental practitioners experience a distinctive type of job demand due to the relationship with their patients [2,7]. Although a harsh work environment including psychosocial job demands is suspected to cause adverse events through practitioners' errors [8-12], there are few studies that have proven this association in the context of medical mismanagement, including dental mismanagement.

The purpose of this study was to examine the association between psychosocial job demands and experiences of adverse events due to dental mismanagement among a group of Japanese dental practitioners. It was hypothesized that dental practitioners exposed to a higher level of job demand would experience adverse events more frequently than those with a lower level of job demand. To date, few studies have implemented questionnaires comprising scales measuring distinctive job demands among dental practitioners [6]. Evidence based on such scales would provide insight into preventive measures against adverse events. In this study, we used the Copenhagen Psychosocial Questionnaire (COPSOQ) – a newly developed, comprehensive instrument to measure psychosocial job demands [13].

## Methods

### Subjects

A self-administered questionnaire was mailed to all members (n = 490) of a local branch of a Japanese dental association in the Kagawa prefecture, Japan (431 men and 59 women). Although this is a small region, the members constituted a representative sample of the dental practitioners of the prefecture. The questionnaire was anonymous. Informed consent was obtained from the respondents by including a question in the questionnaire as to whether they agreed to participate in the study.

### Outcome

An adverse event was defined as any injury or complication in a patient due to dental mismanagement. The outcome was defined according to whether the respondent's patients experienced one of the following adverse events at least once during the previous one year: dropping of dental instrument or broken injection needle, soft tissue or nerve injury (numbness), accidental bleeding, loss of a tooth root into the maxillary sinus, and emphysema.

### Psychosocial job demands

Psychosocial job demands were measured by a Japanese version of the COPSOQ [13]. COPSOQ was developed as a comprehensive tool for assessment and improvement of the psychosocial work environment. The reliability, validity, and applicability of the original Danish version have been discussed [13]. COPSOQ has been translated into several languages. The Japanese version was developed by the translation and back-translation procedure [14]. The Japanese version comprises five sub-scales: quantitative demands (seven items, e.g. "Do you have to work very fast?"), cognitive demands (eight items, e.g. "Does your work require that you remember a lot of things?"), emotional demands (three items, e.g. "Is your work emotionally demanding?"), demands for hiding emotions (two items, e.g. "Does your work require that you hide your feelings?"), and sensorial demands (five items, e.g. "Does your work require a high level of precision?" and "Does your work require that you have to control your movements, e.g. your arms and hands, consciously?"). The scales of the COPSOQ are formed by adding the points of the individual questions, giving equal weight to each question. The questions have five response options and the weights are 0, 25, 50, 75, and 100. The scale value is calculated as the simple average, and all scales range from 0 to 100. Respondents who answer less than half of the questions in a scale are regarded as missing. If a person has answered at least half of the questions, the scale value is calculated as the average of the questions answered. In our study population, Cronbach's alpha coefficients of the scales were 0.88, 0.87, 0.85, 0.65, and 0.82, respectively. In this study, dental practitioners with a score of ≥ 75 points (from 100) in each sub-scale were defined as the group exposed to respective job demands.

The study design and procedure were reviewed and approved by the Human Ethics Committee for Epidemiological Research at the Okayama University Graduate School of Medicine and Dentistry, Japan.

### Statistical analyses

A series of cross-tabulations of sociodemographic and work-related variables with the five job demand indices was performed, using the χ^2 ^test. To examine the association of job demand indices and adverse events experienced, logistic regression analysis was conducted by entering each job stress measure separately. Sex, age (≤ 40, 41–50, 51–60, ≥ 61 years), working hours per week (≤ 40, 41–50, ≥ 51 hours per week), and the numbers of dentists (1, 2, ≥ 3), paramedical staff (≤ 3, 4–5, ≥ 6), patients per day (up to around 25, around 30–35, around 40 or more), and dental chairs in the clinic (≤ 2, 3, 4, ≥ 5) were selected as confounding factors in the logistic models. Ordinal or discrete variables were represented by dummy variables. All associations were inferred with an α level of 0.05. These were performed using the SPSS computer program, version 13 (Chicago, IL, USA).

## Results

A total of 261 dental practitioners responded (response rate 53%). Table 1 shows the profile of the study population. We found no significant differences in the proportions of gender, age groups, and specialties in our study population compared with the total population of the branch (data not shown).

**Table 1:** Profile of the study population (N = 261)

Study variables	%
Sex	
Men	90
Women	10
Age (yeas old)	
≤40	20
41–50	35
51–60	26
≥61	18
Working hours per week	
≤40	40
41–50	45
≥51	15
Numbers of patients per day	
Up to around 25	62
Around 30–35	23
Around 40 or more	15
Numbers of dentists	
1	64
2	30
≥3	7
Number of paramedical staff in the clinic	
≤3	40
4–5	36
≥6	24
Number of the dental chairs in the clinic	
≤2	11
3	44
4	29
≥5	16

The prevalences of experiences of adverse events during the previous year were as follows: soft tissue injury 27%, accidental bleeding 18%, dropping of dental instrument 10%, emphysema 3%, nerve injury (numbness) 2%, dropping of broken injection needle 1%, and loss of a tooth root into the maxillary sinus 1%. Of the respondents, 113 dental practitioners (43%) experienced one of the adverse events at least once during the previous year.

Table 2 shows the relationship between sociodemographic and work conditions and psychosocial job demands. The psychosocial job demands appeared to reflect the hectic work condition of the dentists. Quantitative demands were associated with working hours per week, and the numbers of patients per day, paramedical staff, and dental chairs in the clinic. Cognitive demands were associated with the number of patients, paramedical staff, and dental chairs in the clinic. Cognitive demands were more prevalent among younger dentists than older dentists. Emotional demands were associated with the numbers of patients per day and paramedical staff in the clinic. Sensorial demands were associated with the number of paramedical staff in the clinic. There were no statistically significant associations between demand for hiding emotions and selected work conditions.

**Table 2:** Sociodemographic and work conditions and psychosocial job demands

	Quantitative demands		Cognitive demands		Emotional demands		Demands for hiding emotions		Sensorial demands	
	*%*	*p*	*%*	*p*	*%*	*p*	*%*	*p*	*%*	*p*

Sex										
Men	12	0.585	47	0.940	9	0.681	13	0.855	91	0.394
Women	8		48		12		12		96	
Age (yeas old)										
≤40	19	0.070	64	0.008	12	0.162	14	0.223	89	0.892
41–50	11		51		14		17		91	
51–60	12		40		6		15		93	
≥61	2		32		4		4		92	
Working hours per week										
≤40	5	<0.001	44	0.068	7	0.046	10	0.120	91	0.606
41–50	10		44		9		13		90	
≥51	33		64		21		23		95	
Numbers of patients per day										
Up to around 25	5	<0.001	37	<0.001	5	0.005	11	0.105	88	0.088
Around 30–35	19		54		14		12		95	
Around 40 or more	26		74		21		24		97	
Numbers of dentists										
1	12	0.800	47	0.302	8	0.332	14	0.644	92	0.804
2	9		44		12		13		90	
≥3	12		65		18		6		94	
Number of paramedical staff in the clinic										
≤3	5	0.005	30	<0.001	5	0.001	13	0.732	87	0.017
4–5	10		53		7		12		90	
≥6	22		67		22		17		100	
Number of the dental chairs in the clinic										
≤2	4	0.040	38	0.008	3	0.104	7	0.511	79	0.103
3	7		38		6		13		93	
4	19		60		15		18		91	
≥5	15		59		15		12		95	

The associations between psychosocial job demands and experience of adverse events due to dental mismanagement are shown in Table 3. Except for 'Demands for hiding emotions,' dental practitioners exposed to higher levels of job demands had higher odd ratios of experiences of adverse events due to dental mismanagement. Of the psychosocial job demand indices, emotional and sensorial demands were significantly associated with the experience of adverse events (odds ratio = 3.9 for each).

**Table 3:** Psychosocial demands and experience of adverse events due to dental mismanagement

Psychosocial demands	Adjusted odds ratio	95% confidence interval
Quantitative demands	1.5	0.5, 2.4
Cognitive demands	1.4	0.8, 2.6
Emotional demands	3.9	1.3, 12.1
Demands for hiding emotions	1.0	0.4, 2.2
Sensorial demands	3.9	1.1, 13.3

Notes: Logistic models were employed by entering each job demand measure separately. Adjusted for sex, age, working hours per week, and the numbers of patients per day, dentists, paramedical staff, and the dental chairs in the clinic.

Other than the job demand indices, males, dentists younger than 40 years, single-handed, 30 or more patients per day, and five or more dental chairs were identified as being high risk for adverse events (data not shown). Working hours per week and the number of paramedical staff had no significant associations with adverse events due to dental mismanagement.

## Discussion

Using a newly validated comprehensive job demands questionnaire, this cross-sectional study of dental practitioners revealed that two types of psychosocial job demand – emotional and sensorial – were associated with experiences of adverse events due to dental mismanagement. Since research suggests that stress management programs lead to a reduction in medical malpractice incidents [15], our findings imply that the indicated job demands are an important target for the reduction of adverse events due to dental mismanagement.

It is possible that clinics with more beds or more patient contact hours may have higher demand levels and are more likely to be at risk of medical errors or malpractice. Even after adjusting for these possible confounders, emotional and sensorial demands were independently associated with experiences of adverse events due to dental mismanagement. There is empirical evidence that the type of job demand is associated with occupational accidents or injuries. Swaen et al. observed that high psychological job demands, emotional demands, and conflicts with the supervisor and/or colleagues were risk factors for being injured in an occupational accident [12]. Experiencing a high degree of ergonomic stress was reported to be a risk factor for occupational injuries [16]. Too many such demands might lead to distress, fatigue, or poor cognitive performance, including difficulty in giving constant care or impaired health care judgments and decision-making among dental practitioners [17,18], which in turn increase the risk of errors.

Other than the psychosocial job demand indices, conditions of the workplace such as the number of patients per day and the number of dentists were also associated with experienced adverse events. A previous study revealed that dentists were more likely to report high levels of emotional exhaustion and low levels of personal accomplishment if they worked in practices with few other dentists [19]. Furthermore, social support in the workplace, measured here by the number of dentists in the practice, appears to have a protective effect against some aspects of burnout [19]. Similarly, a survey in general practitioners revealed that having little free time from practice work was associated with depression [20]. Lack of resources (help) and the consequences might be associated with adverse events due to dental mismanagement.

The demographic characteristics that were shown to be associated with adverse events due to dental mismanagements seem to be compatible with the related literature. Employees who were injured in an occupational accident were more likely to be male [12]. Younger workers tend to have higher occupational accident rates than older workers [12,21]. The number of patients per day was associated with adverse events, but working hours were not. Rather than just the length of working hours, the intensity of demands might be a more important quantitative predictor of occupational accidents [13].

Measurement of distinctive job demands would allow us to consider concrete preventive measures of adverse events due to dental mismanagement. Both emotional and sensorial demands tap the psychological aspect rather than environmental or organizational. Individual approaches, including coping or relaxation, would be an appropriate measure to counteract the consequences of such demands. Ergonomic measures, such as a supporting devices for the arms, are also applicable against sensorial demands. Furthermore, the findings suggest the importance of fostering a supportive environment.

There are some limitations of our study. Firstly, the subjects were limited to members of a local branch of a Japanese dental association, thus restricting the applicability of the results to the general population. Furthermore, as the response rate of this study was slightly low, we cannot deny that dental practitioners who were extremely busy and those who had perpetrated medical errors might have not responded to the questionnaire or under-reported their experiences, which could lead to underestimation of the associations. However, attrition analysis supported the view that our study population was representative of the dental practitioners of the prefecture. Secondly, the present study was a cross-sectional study using a self-administered questionnaire, and thus a causal relationship between psychosocial job demands and experienced adverse events could not be determined. High levels of job demands may have led to a high incidence of adverse events, but it is also possible that dental practitioners with a history of adverse events may pay more attention to their working situation; that is, they may perceive greater job demands. However, the reverse association, such as increasing patient numbers after adverse events, is less likely.

## Conclusion

Despite the above-mentioned limitations, our study sheds light on the importance of psychosocial job demands as a target for the prevention of adverse events due to dental mismanagement. More specifically, coping against emotional and sensorial demands could be a worthwhile strategy. The implications of our study also include the observation that enhanced support resources within the workplace could be effective in reducing adverse events.

## Competing interests

The author(s) declare that they have no competing interests.

## Authors' contributions

AT conceived of the study, and participated in its design and coordination and drafted the manuscript. KU performed the statistical analysis and helped to draft the manuscript. HO participated in the design of the study and helped to collect the data and draft the manuscript. NK participated in coordination of the study and helped to draft the manuscript. All authors read and approved the final manuscript.

## Pre-publication history

The pre-publication history for this paper can be accessed here:


